# Inhibition of bone erosion, determined by high-resolution peripheral quantitative computed tomography (HR-pQCT), in rheumatoid arthritis patients receiving a conventional synthetic disease-modifying anti-rheumatic drug (csDMARD) plus denosumab vs csDMARD therapy alone: an open-label, randomized, parallel-group study

**DOI:** 10.1186/s13075-022-02957-w

**Published:** 2022-12-07

**Authors:** Naoki Iwamoto, Ko Chiba, Shuntaro Sato, Kazuteru Shiraishi, Kounosuke Watanabe, Nozomi Oki, Akitomo Okada, Tomohiro Koga, Shin-ya Kawashiri, Mami Tamai, Naoki Hosogaya, Masako Furuyama, Makiko Kobayashi, Kengo Saito, Naoki Okubo, Masataka Uetani, Makoto Osaki, Atsushi Kawakami

**Affiliations:** 1 Department of Immunology and Rheumatology, Division of Advanced Preventive Medical Sciences, Nagasaki University Graduate School of Biomedical Sciences, 1-7-1 Sakamoto, Nagasaki, 852-8501 Japan; 2 Department of Orthopedic Surgery, Nagasaki University Graduate School of Biomedical Sciences, 1-7-1 Sakamoto, Nagasaki, 852-8501 Japan; 3 Clinical Research Center, Nagasaki University Hospital, 1-7-1 Sakamoto, Nagasaki, 852-8501 Japan; 4 Department of Radiological Sciences, Nagasaki University Graduate School of Biomedical Sciences, 1-7-1 Sakamoto, Nagasaki, 852-8501 Japan; 5 Department of Rheumatology, National Hospital Organization Nagasaki Medical Center, 2-1001-1 Kubara, Omura, Nagasaki, 856-8562 Japan; 6 Departments of Community Medicine, Division of Advanced Preventive Medical Sciences, Nagasaki University Graduate School of Biomedical Sciences, 1-7-1 Sakamoto, Nagasaki, 852-8501 Japan; 7 Department of Rheumatology, Nagasaki Kita Hospital, 800 Motomurago, Nishisonogigun Togitsucho, Nagasaki, 851-2103 Japan; 8 Primary Medical Science Department, Medical Affairs Division, Daiichi Sankyo Co., Ltd, 3-5-1 Nihonbashi-Honcho, Chuo-ku, Tokyo, 103-8426 Japan; 9 Data Intelligence Department, Digital Transformation Management Division, Daiichi Sankyo Co., Ltd, 1-2-58, Hiromachi, Shinagawa-ku, Tokyo, 140-8710 Japan

**Keywords:** Bone erosion, csDMARDs, Denosumab, HR-pQCT, Rheumatoid arthritis

## Abstract

**Background:**

This exploratory study compared the inhibition of bone erosion progression in rheumatoid arthritis (RA) patients treated with a conventional synthetic disease-modifying anti-rheumatic drug (csDMARD) plus denosumab versus csDMARD therapy alone and investigated the effects of denosumab on bone micro-architecture and other bone-related parameters using high-resolution peripheral quantitative computed tomography (HR-pQCT).

**Methods:**

In this open-label, randomized, parallel-group study, patients with RA undergoing treatment with a csDMARD were randomly assigned (1:1) to continue csDMARD therapy alone or to continue csDMARDs with denosumab (60-mg subcutaneous injection once every 6 months) for 12 months. The primary endpoint was the change from baseline in the depth of bone erosion, measured by HR-pQCT, in the second and third metacarpal heads at 6 months after starting treatment. Exploratory endpoints were also evaluated, and adverse events (AEs) were monitored for safety.

**Results:**

In total, 46 patients were enrolled, and 43 were included in the full analysis set (csDMARDs plus denosumab, *N* = 21; csDMARD therapy alone, *N* = 22). Most patients were female (88.4%), and the mean age was 65.3 years. The adjusted mean (95% confidence interval) change from baseline in the depth of bone erosion, measured by HR-pQCT, in the 2–3 metacarpal heads at 6 months was − 0.57 mm (− 1.52, 0.39 mm) in the csDMARDs plus denosumab group vs − 0.22 mm (− 0.97, 0.53 mm) in the csDMARD therapy alone group (between-group difference: − 0.35 mm [− 1.00, 0.31]; *P* = 0.2716). Similar results were shown for the adjusted mean between-group difference in the width and volume of bone erosion of the 2–3 metacarpal heads. Significant improvements in bone micro-architecture parameters were shown. The incidence of AEs and serious AEs was similar between the csDMARDs plus denosumab and the csDMARD therapy alone groups (AEs: 52.2% vs 56.5%; serious AEs: 4.3% vs 8.7%).

**Conclusions:**

Although the addition of denosumab to csDMARDs did not find statistically significant improvements in bone erosion after 6 months of treatment, numerical improvements in these parameters suggest that the addition of denosumab to csDMARDs may be effective in inhibiting the progression of bone erosion and improving bone micro-architecture.

**Trial registration:**

University Hospital Medical Information Network Clinical Trials Registry, UMIN000030575. Japan Registry for Clinical Trials, jRCTs071180018

**Supplementary Information:**

The online version contains supplementary material available at 10.1186/s13075-022-02957-w.

## Background

Rheumatoid arthritis (RA) is a chronic, progressive, inflammatory autoimmune disease that is characterized by persistent inflammation of the synovial membrane (synovitis) [1–3]. Clinical symptoms include pain, swelling, stiffness of multiple joints, fever, and malaise. Bone erosions develop early in the course of this disease, leading to irreversible joint damage and physical disability.

Denosumab is a fully human monoclonal immunoglobulin G2 antibody specific to the receptor activator of nuclear factor-κB ligand (RANKL) that suppresses bone resorption by competitive inhibition of RANKL [4]. RANKL-RANK signaling is essential for osteoclast development, activation, and survival [5]. Clinical research has shown that denosumab is effective in increasing bone mineral density (BMD) and reducing fragility fractures in patients with osteoporosis [6]. Denosumab has also been shown to be effective in suppressing bone destruction in patients with RA [7–9].

The DESIRABLE study was a multicenter, randomized, double-blind, placebo-controlled phase 3 trial conducted in Japan to evaluate the efficacy of denosumab in suppressing joint destruction when added to a conventional synthetic disease-modifying anti-rheumatic drug (csDMARD) in patients with RA [9]. Dual-energy X-ray absorptiometry of the lumbar spine and hand and foot X-ray indicated that denosumab significantly increased BMD and inhibited the progression of bone erosion (an important indicator of disease severity and progression) in RA patients after 12 months of treatment.

In 2017, denosumab was approved in Japan for the indication of inhibiting the progression of bone erosion associated with RA [10]. Although the effectiveness and safety of denosumab in patients with RA have been established from clinical trial data [7–9], there remain clinical questions to be explored, such as the prognosis after treatment with denosumab in clinical practice in terms of changes in joint destruction and periarticular bone micro-architecture.

High-resolution peripheral quantitative computed tomography (HR-pQCT) is a relatively new technique that uses a three-dimensional imaging modality [11]. HR-pQCT achieves a higher spatial resolution than conventional methods such as X-ray, CT, or magnetic resonance imaging (MRI), with the advantage of relatively low radiation exposure, and allows the assessment of the bone micro-architecture, which cannot be evaluated with conventional methods. Of note, this imaging modality allows for a quantitative evaluation of bone erosion by measuring the depth, volume, and width of bone erosion at peripheral sites [12]. To date, the use of HR-pQCT in clinical trials of denosumab has been limited [13].

The primary objective of this study was to compare the inhibition of bone erosion progression in RA patients treated with a csDMARDs plus denosumab versus csDMARD therapy alone [14]. The secondary objectives were to investigate the effects of denosumab on bone micro-architecture, osteitis, joint destruction, and periarticular osteoporosis in RA patients using HR-pQCT and to examine the effect of denosumab on bone erosion independent of inflammation.

## Methods

### Study design

Details of the study design and protocol have been described previously [14]. This was an exploratory, open-label, randomized, parallel-group study, which was conducted from March 2018 to April 2021. Although the study was originally planned to be conducted at a single site, a decision was made in September 2019 to expand this to a multicenter study. RA patients undergoing treatment with a csDMARD were randomly assigned (1:1) to continue csDMARD therapy and either receive additional treatment with denosumab (csDMARDs plus denosumab group) or to continue with csDMARDs alone (csDMARD therapy alone group) for a period of 12 months. The allocation method used was the minimization method, and random allocation was performed automatically on the allocation system. The allocation factors were the presence or absence of anti-cyclic citrullinated peptide (CCP) antibodies and sex.

The study protocol was approved by the Nagasaki University Hospital Clinical Research Ethics Committee and the Clinical Research Review Board in Nagasaki University, and the study was conducted in accordance with the principles of the Declaration of Helsinki and Clinical Trials Act (since February 2019). All patients provided written informed consent. This study was registered at the University Hospital Medical Information Network Clinical Trials Registry under the identifier UMIN000030575 and at the Japan Registry for Clinical Trials under the identifier jRCTs071180018.

### Patients

Patients who fulfilled all of the following criteria were included in this study: age ≥ 20 years, with a diagnosis of RA based on the American College of Rheumatology (ACR) RA Classification Criteria 1987 Revision or ACR/European Alliance of Associations for Rheumatology (EULAR) 2010 RA Classification Criteria [15, 16], with low to moderate disease activity as defined by the Disease Activity Score in 28 joints for RA with erythrocyte sedimentation rate (DAS28-ESR), receiving treatment with any csDMARD, and with progressive bone erosion of the wrist or any metacarpal head (not limited to the 2–3 metacarpal heads) confirmed by X-ray, MRI, or musculoskeletal ultrasound imaging.

The main exclusion criteria were as follows: patients with osteoporosis who had not received treatment for osteoporosis; patients undergoing treatment with intravenous bisphosphonate, parathyroid hormone analog, denosumab, any biologic DMARD, a JAK inhibitor, or a corticosteroid equivalent of more than 10 mg/day of prednisolone; hypersensitivity to denosumab or any of its components; patients with hypocalcemia; pregnant patients; and those judged as inappropriate by the investigator to participate in the study.

### Treatment

Patients in the csDMARDs plus denosumab group received 60-mg denosumab administered by subcutaneous injection once every 6 months during the 12-month study period, and daily oral vitamin D and calcium supplements were also initiated. Patients in this group who had received any oral bisphosphonate or selective estrogen receptor modulator before study entry were to discontinue these drugs before initiating treatment with denosumab. Patients in the csDMARD therapy alone group continued their treatment throughout the study period. In this group, patients continued oral bisphosphonate or selective estrogen receptor modulator during the study. All study participants were to continue treatment with at least one csDMARD throughout the study period. Switching from one csDMARD to another, adding a new csDMARD, discontinuing a part of the csDMARD therapy, and modifying the dose of csDMARD therapy within the dose range approved in Japan were allowed during the study period. Additional details of the study treatment have been described previously [14].

### Efficacy endpoints

The primary endpoint was the change from baseline in the depth of bone erosion as measured by HR-pQCT in the 2–3 metacarpal heads at 6 months after starting treatment. Secondary endpoints included changes from baseline in the width and volume of bone erosion as measured by HR-pQCT in the 2–3 metacarpal heads and changes from baseline in the depth of bone erosion in the 2–3 metacarpal heads at 12 months.

### Exploratory endpoints

Disease activity was assessed by the DAS28-ESR, and joint destruction was assessed by MRI and HR-pQCT. The following exploratory endpoints were evaluated: using HR-pQCT, changes from baseline in periarticular volumetric BMD (vBMD) and bone micro-architecture of the 2–3 metacarpal heads; using MRI, changes from baseline in the extent of osteitis (osteitis score) in the metacarpal head and wrist joints and change from baseline in the extent of bone erosion (bone erosion score); using musculoskeletal ultrasound, changes from baseline in synovitis score; using X-ray measurements, change from baseline in bone erosion score, joint space narrowing (JSN) score, modified total Sharp score (mTSS), and joint destruction score (the sum of erosion and JSN scores of the 2–3 metacarpal heads); change from baseline in DAS28-ESR; and change from baseline in bone and cartilage biomarkers, including procollagen type I N-terminal propeptide, tartrate-resistant acid phosphatase-5b, and matrix metalloproteinase-3.

### Safety endpoints

The incidence of adverse events (AEs), serious AEs, and adverse drug reactions (ADRs) was evaluated. ADRs were defined as AEs that were judged to be “related” to denosumab in a causal relationship. AEs were coded by System Organ Class and Preferred Term using the Japanese version of the Medical Dictionary for Regulatory Activities, version 23.1.

### Statistical methods

Sample size calculations have been described previously [14]. The target sample size was set at 44 patients (22 patients per cohort). In accordance with the intention-to-treat principle, efficacy was assessed using the full analysis set (FAS), defined as all enrolled patients with HR-pQCT measurements available both at baseline and 6 months. Safety was evaluated using the safety analysis set, defined as all patients who were randomly assigned to treatment.

The number and percentage of patients were calculated for categorial variables, and the mean and standard deviation were calculated for continuous data. For the primary endpoint, a linear mixed effect model analysis was performed using treatment group, baseline values of the endpoint, sex, anti-CCP antibody (positive vs negative), and baseline disease activity (DAS28-ESR) as fixed effects and patients as random effects. For extension data including 12 months, measurement time point and the interaction between the treatment group and measurement time point were added as fixed effects. The model estimation method was the restricted maximum likelihood estimation, the covariance structure was unstructured, and the calculation method for the degree of freedom was the Kenward–Roger. Adjusted mean change from baseline by time point, the differences of adjusted mean change, and 95% confidence intervals (CI) for the above models were estimated.

For exploratory endpoints, descriptive statistics were calculated for measured values in each treatment group and observation point using the FAS as the analysis population. In addition, the adjusted mean, the difference, and the 95% CIs for each treatment group and each time point were estimated using the same model as above. *P* values < 0.05 were considered significant. All statistical analyses were performed using SAS software version 9.4 (SAS Institute Inc., Cary, NC, USA).

## Results

### Patients

**Fig. 1:**
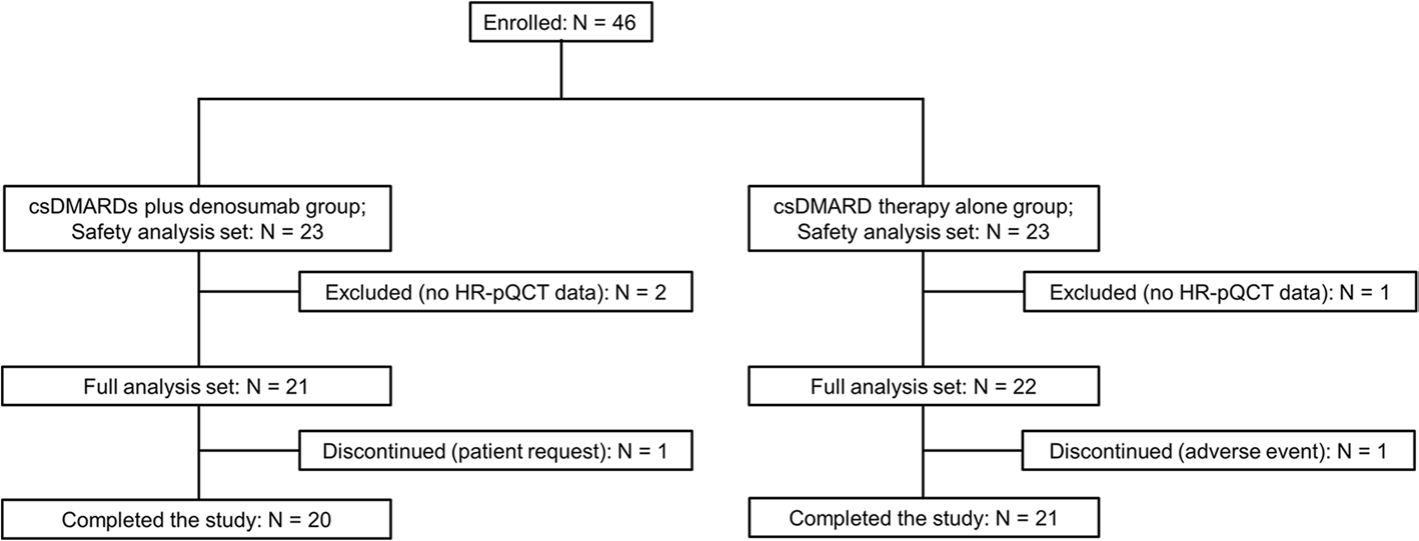
Patient disposition. csDMARD, conventional synthetic disease-modifying anti-rheumatic drug; HR-pQCT, high-resolution peripheral quantitative computed tomography

A total of 46 patients were enrolled and included in the safety analysis set (23 patients in each group). Two patients were excluded from the csDMARDs plus denosumab group, and one patient was excluded from the csDMARD therapy alone group because they did not have HR-pQCT measurements available at either 0 or 6 months. Therefore, 43 patients were included in the FAS (csDMARDs plus denosumab group, *N* = 21; csDMARD therapy alone group, *N* = 22) (Fig. 1). One patient in the csDMARDs plus denosumab group discontinued the study due to the patient’s own request to change or discontinue treatment, and one patient in the csDMARD therapy alone group discontinued the study due to an AE.

Overall, patient background characteristics were similar between the groups (Table 1). Most patients were female (88.4%), the mean age was 65.3 ± 8.8 years, the mean duration of RA was 156.4 ± 139.0 months, and most patients had a duration of RA ≥ 3 years (81.0%). Ten patients had a history of fracture, and the fracture sites as events were one vertebra, one rib, and four other sites in the csDMARDs plus denosumab group and two humeri, one radius, and three other sites in the csDMARD therapy alone group. More than half of the patients (60.5%) had osteoporosis as a complication. The proportions of patients with erosions of the 2–3 metacarpal heads were 38.1% (8/21) in the csDMARDs plus denosumab group and 59.1% (13/22) in the csDMARD therapy alone group. Of note, the proportion of patients who used a glucocorticoid was 47.6% (10/21) in the csDMARDs plus denosumab group compared with 18.2% (4/22) in the csDMARD therapy alone group and that of patients who used bisphosphonates was 0% and 18.2% (4/22), respectively. The distribution of csDMARD use also differed between the two groups. Methotrexate and salazosulfapyridine were used more frequently in the csDMARDs plus denosumab group (90.5% and 33.3%, respectively) compared with the csDMARD therapy alone group (72.7% and 13.6%, respectively). Conversely, tacrolimus hydrate, iguratimod, and bucillamine were used more frequently in the csDMARD therapy alone group (13.6%, 9.1%, and 13.6%, respectively) compared with the csDMARDs plus denosumab group (4.8%, 4.8%, and 0%, respectively).

**Table 1:** Baseline patient demographic and clinical characteristics (full analysis set)

	csDMARDs plus denosumab (*N* = 21)	csDMARD therapy alone (*N* = 22)
Female sex	18 (85.7)	20 (90.9)
Age, years	65.6 ± 8.5	65.0 ± 9.2
≥ 65 years	12 (57.1)	11 (50.0)
Body mass index, kg/m^2^	22.94 ± 3.42	22.64 ± 4.48
Duration of rheumatoid arthritis, months	162.3 ± 151.3	150.5 ± 129.0
< 6 months	1 (4.8)	0 (0.0)
6 to < 36 months	4 (19.0)	3 (14.3)
≥ 36 months	16 (76.2)	18 (85.7)
Osteoporosis	15 (71.4)	11 (50.0)
Prior fracture	5 (23.8)	5 (22.7)
Medical history other than fracture		
Knee arthroplasty	1 (4.8)	0 (0.0)
Tonsillectomy	1 (4.8)	0 (0.0)
Bone erosion (HR-pQCT of 2–3 metacarpal heads)	8 (38.1)	13 (59.1)
Anti-CCP antibody-positive	16 (76.2)	17 (77.3)
Rheumatoid factor-positive	17 (81.0)	18 (81.8)
Concomitant medications		
Vitamin D and calcium	21 (100.0)	14 (63.6)
Bisphosphonates	0 (0.0)	4 (18.2)
Glucocorticoids	10 (47.6)	4 (18.2)
csDMARDs	21 (100.0)	22 (100.0)
Methotrexate	19 (90.5)	16 (72.7)
Salazosulfapyridine	7 (33.3)	3 (13.6)
Tacrolimus hydrate	1 (4.8)	3 (13.6)
Iguratimod	1 (4.8)	2 (9.1)
Bucillamine	0 (0.0)	3 (13.6)
DAS28-ESR	3.064 ± 0.834	3.041 ± 0.879

Data are shown as *n* (%) or mean ± SD

*CCP* cyclic citrullinated peptide, *csDMARD* conventional synthetic disease-modifying anti-rheumatic drug, *DAS28-ESR* Disease Activity Score in 28 joints for rheumatoid arthritis with erythrocyte sedimentation rate, *HR-pQCT* high-resolution peripheral quantitative computed tomography, *SD* standard deviation

### Efficacy endpoints

**Fig. 2:**
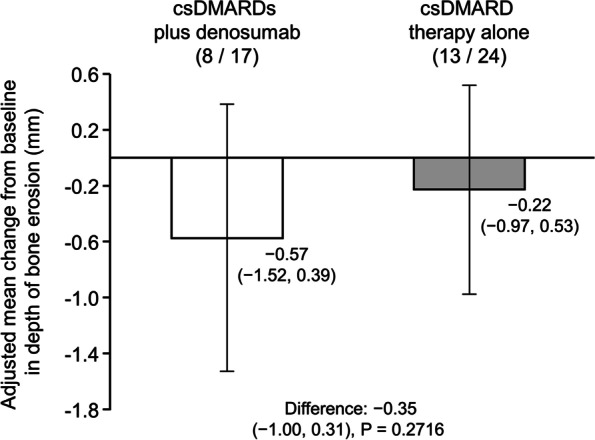
Adjusted mean change from baseline in bone erosion depth of the 2–3 metacarpal heads at 6 months. Assessed by HR-pQCT (primary endpoint, full analysis set). Error bars represent 95% CI. Numerators reflect the number of patients with available data on erosion parameters, and denominators, the number of bone erosions. *P* value is the statistical difference in the csDMARDs plus denosumab vs csDMARD therapy alone group. For adjusted mean as the primary endpoint, a linear mixed effect model analysis was performed using treatment group, sex, anti-CCP antibody (positive vs negative), and baseline DAS28-ESR as fixed effects; patients as random effects; and baseline values as covariates. CCP, cyclic citrullinated peptide; CI, confidence interval; csDMARD, conventional synthetic disease-modifying anti-rheumatic drug; DAS28-ESR, Disease Activity Score in 28 joints for rheumatoid arthritis with erythrocyte sedimentation rate as fixed effects; HR-pQCT, high-resolution peripheral quantitative computed tomography

The adjusted mean change from baseline in the depth of bone erosion as measured by HR-pQCT in the 2–3 metacarpal heads at 6 months after starting treatment (primary endpoint) was − 0.57 mm (95% CI: − 1.52, 0.39 mm) in the csDMARDs plus denosumab group vs − 0.22 mm (95% CI: − 0.97, 0.53 mm) in the csDMARD therapy alone group (Fig. 2 and Additional file 1: Table S1). The adjusted mean difference in the primary endpoint between the two groups was − 0.35 mm (95% CI: − 1.00, 0.31; *P* = 0.2716).

The results of the secondary endpoints are summarized in Table 2. As mentioned above, the statistical methods for the adjusted mean were different for the primary and secondary endpoints. Similar results were shown for the adjusted mean difference between the two groups in the depth (change from baseline to month 12: − 0.35 mm 95% CI: − 0.95, 0.24; *P* = 0.2251), width (change from baseline to month 6: − 0.20 mm, 95% CI: − 0.81, 0.40; *P* = 0.4953; change from baseline to month 12: − 0.24 mm, 95% CI: − 0.85, 0.38; *P* = 0.4364), and volume (change from baseline to month 6: − 4.51 mm^3^, 95% CI: − 16.67, 7.65; *P* = 0.4379; change from baseline to month 12: − 3.07 mm^3^, 95% CI: − 15.32, 9.17; *P* = 0.5979) of bone erosion in the 2–3 metacarpal heads.

**Table 2:** Change from baseline in bone erosion parameters evaluated by HR-pQCT (full analysis set)

	Month	csDMARDs plus denosumab (***N*** = 21)		csDMARD therapy alone (***N*** = 22)		Difference (csDMARDs plus denosumab − csDMARD therapy alone)
		***n***	Adjusted mean [95% CI]	***n***	Adjusted mean [95% CI]	Adjusted mean [95% CI] ***P*** value
**Bone erosion depth of the 2–3 metacarpal heads, mm**	6	17	− 0.46 [− 1.31, 0.39]	25	− 0.20 [− 0.89, 0.49]	− 0.27 [− 0.86, 0.32] *P* = 0.3486
	12	17	− 0.56 [− 1.41, 0.29]	22	− 0.20 [− 0.90, 0.49]	− 0.35 [− 0.95, 0.24] *P* = 0.2251
**Bone erosion width of the 2–3 metacarpal heads, mm**	6	17	− 0.26 [− 1.10, 0.57]	25	− 0.06 [− 0.73, 0.61]	− 0.20 [− 0.81, 0.40] *P* = 0.4953
	12	17	− 0.27 [− 1.10, 0.56]	22	− 0.03 [− 0.70, 0.64]	− 0.24 [− 0.85, 0.38] *P* = 0.4364
**Bone erosion volume of the 2–3 metacarpal heads, mm**^**3**^	6	16	− 6.21 [− 23.89, 11.46]	24	− 1.71 [− 16.07, 12.66]	− 4.51 [− 16.67, 7.65] *P* = 0.4379
	12	17	− 6.25 [− 23.94, 11.44]	21	− 3.18 [− 17.56, 11.20]	− 3.07 [− 15.32, 9.17] *P* = 0.5979

Data are shown as adjusted mean [95% CI] unless otherwise indicated

*n* is the number of bone erosions

For adjusted mean, a linear mixed effect model analysis was performed using treatment group, sex, anti-CCP antibody (positive vs negative), baseline DAS28-ESR, measurement time point, and the interaction between the treatment group and measurement time point as fixed effects; patients as random effects; and baseline values as covariates

*CCP* cyclic citrullinated peptide, *CI* confidence interval, *csDMARD* conventional synthetic disease-modifying anti-rheumatic drug, *DAS28-ESR* Disease Activity Score in 28 joints for rheumatoid arthritis with erythrocyte sedimentation rate as fixed effects, *HR-pQCT* high-resolution peripheral quantitative computed tomography

### Exploratory endpoints

The difference in the percentage change in periarticular vBMD of the 2–3 metacarpal heads by HR-pQCT from baseline to month 12 between the csDMARDs plus denosumab group and the csDMARD therapy alone group was 4.03% (95% CI: 1.28, 6.78, *P* = 0.0049) (Table 3). Similar results were obtained in trabecular bone volume fraction (BV/TV) (difference in % change 3.10%, 95% CI: 0.97, 5.22; *P* = 0.0050), trabecular thickness (Tb.Th) (difference in % change 1.57%, 95% CI: 0.06, 3.09; *P* = 0.0421), and trabecular separation (Tb.Sp) (difference in % change − 4.14%, 95% CI: − 8.07, − 0.20; *P* = 0.0397).

**Table 3:** Change from baseline in bone micro-architecture parameters evaluated by HR-pQCT (full analysis set)

	Month	csDMARDs plus denosumab (***N*** = 21)		csDMARD therapy alone (***N*** = 22)		Difference (csDMARDs plus denosumab − csDMARD therapy alone)
		***n***	Adjusted mean [95% CI]	***n***	Adjusted mean [95% CI]	Adjusted mean [95% CI] ***P*** value
**vBMD of the 2–3 metacarpal heads, %**	6	42	4.57 [2.12, 7.03]	44	2.17 [− 0.39, 4.74]	2.40 [− 0.29, 5.10] *P* = 0.0791
	12	38	6.90 [4.37, 9.43]	42	2.87 [0.29, 5.45]	4.03 [1.28, 6.78] *P* = 0.0049
**BV/TV of the 2–3 metacarpal heads, %**	6	42	3.88 [1.98, 5.77]	44	1.77 [− 0.21, 3.76]	2.11 [0.03, 4.19] *P* = 0.0473
	12	38	5.41 [3.45, 7.36]	42	2.31 [0.31, 4.31]	3.10 [0.97, 5.22] *P* = 0.0050
**Tb.Th of the 2–3 metacarpal heads, %**	6	42	1.51 [0.24, 2.78]	44	0.51 [– 0.81, 1.83]	1.00 [− 0.47, 2.46] *P* = 0.1799
	12	38	2.37 [1.02, 3.71]	42	0.80 [− 0.53, 2.12]	1.57 [0.06, 3.09] *P* = 0.0421
**Tb.Sp of the 2–3 metacarpal heads, %**	6	42	− 2.46 [− 5.89, 0.98]	44	0.38 [− 3.15, 3.92]	− 2.84 [− 6.67, 0.99] *P* = 0.1431
	12	38	− 2.18 [− 5.78, 1.41]	42	1.95 [− 1.61, 5.51]	− 4.14 [− 8.07, − 0.20] *P* = 0.0397

Data are shown as adjusted mean [95% CI] unless otherwise indicated

*n* is the number of joints evaluated

For adjusted mean, a linear mixed effect model analysis was performed using treatment group, sex, anti-CCP antibody (positive vs negative), baseline DAS28-ESR, measurement time point, and the interaction between the treatment group and measurement time point as fixed effects; patients as random effects; and baseline values as covariates

*BV/TV* trabecular bone volume fraction, CCP cyclic citrullinated peptide, *CI* confidence interval, *csDMARD* conventional synthetic disease-modifying anti-rheumatic drug, *DAS28-ESR* Disease Activity Score in 28 joints for rheumatoid arthritis with erythrocyte sedimentation rate, *HR-pQCT* high-resolution peripheral quantitative computed tomography, *Tb.Sp* trabecular separation, *Tb.Th* trabecular thickness, *vBMD* volumetric bone mineral density

The percent change values of other exploratory endpoints evaluated by MRI, musculoskeletal ultrasound, and X-ray are summarized in Table 4 and Additional file 1: Table S2. The between-group difference in the percentage change in total osteitis score by MRI from baseline to month 12 was 26.04% (95% CI: − 51.02, 103.09; *P* = 0.5006); bone erosion score by MRI, − 6.65% (95% CI: − 26.65, 13.35; *P* = 0.5085); and power Doppler (PD) score by musculoskeletal ultrasound, 12.82% (95% CI: − 74.02, 99.67; *P* = 0.7645). Regarding the changes from baseline in bone erosion scores by X-ray, the results showed that bone erosion was suppressed at both months 6 and 12 in the csDMARDs plus denosumab group compared with the csDMARD therapy alone group, although the differences were not statistically significant. These results were consistent with the HR-pQCT results.

**Table 4:** Change from baseline in other exploratory endpoints evaluated by MRI, musculoskeletal ultrasound, and X-ray (full analysis set)

	Month	csDMARDs plus denosumab (***N*** = 21)		csDMARD therapy alone (***N*** = 22)		Difference (csDMARDs plus denosumab − csDMARD therapy alone)
		***N***	Adjusted mean [95% CI]	***N***	Adjusted mean [95% CI]	Adjusted mean [95% CI] ***P*** value
**Total osteitis score of the metacarpal heads and wrist joints by MRI, %**	6	17	7.45 [− 55.95, 70.86]	18	28.87 [− 38.70, 96.44]	− 21.42 [− 97.14, 54.30] *P* = 0.5725
	12	16	47.79 [− 18.34, 113.92]	17	21.75 [− 46.33, 89.84]	26.04 [− 51.02, 103.09] *P* = 0.5006
**Bone erosion score of the metacarpal heads and wrist joints by MRI, %**	6	18	4.49 [− 12.67, 21.64]	19	5.16 [− 11.69, 22.02]	− 0.68 [− 19.81, 18.45] *P* = 0.9438
	12	16	13.42 [− 4.89, 31.72]	18	20.07 [3.04, 37.09]	− 6.65 [− 26.65, 13.35] *P* = 0.5085
**PD score in both hands by musculoskeletal ultrasound, %**	6	14	37.61 [− 36.73, 111.95]	15	41.50 [− 36.00, 118.99]	− 3.88 [− 90.12, 82.35] *P* = 0.9271
	12	13	36.57 [− 39.34, 112.48]	15	23.75 [− 53.75, 101.24]	12.82 [− 74.02, 99.67] *P* = 0.7645
**mTSS by X-ray, %**	6	19	− 1.66 [− 15.89, 12.57]	20	2.51 [− 11.53, 16.55]	− 4.17 [− 20.16, 11.82] *P* = 0.6044
	12	18	10.05 [− 5.05, 25.14]	19	7.97 [− 6.24, 22.18]	2.08 [− 14.42, 18.57] *P* = 0.8024

Data are shown as adjusted mean [95% CI] unless otherwise indicated

*N* is the number of patients with available data

For adjusted mean, a linear mixed effect model analysis was performed using treatment group, sex, anti-CCP antibody (positive vs negative), baseline DAS28-ESR, measurement time point, and the interaction between the treatment group and measurement time point as fixed effects; patients as random effects; and baseline values as covariates

*CCP* cyclic citrullinated peptide, *CI* confidence interval, *csDMARD* conventional synthetic disease-modifying anti-rheumatic drug, *DAS28-ESR* Disease Activity Score in 28 joints for rheumatoid arthritis with erythrocyte sedimentation rate, *MRI* magnetic resonance imaging, *mTSS* modified total Sharp score, *PD* power Doppler

The actual values of bone micro-architecture parameters evaluated by HR-pQCT, MRI, musculoskeletal ultrasound, and X-ray are shown in Additional file 1: Table S3. The adjusted mean change in the bone erosion score of total 2–3 metacarpal heads and wrist joints by MRI from baseline to months 6 and 12 was − 0.7 (95% CI: − 2.1, 0.7; *P* = 0.2993) and − 0.4 (95% CI: − 1.9, 1.0; *P* = 0.5680), respectively. The adjusted mean change in total number of bone erosions at both hands by musculoskeletal ultrasound from baseline to months 6 and 12 was − 0.4 (95% CI: − 0.9, 0.2; *P* = 0.2030) and − 0.6 (95% CI: − 1.2, 0.0; *P* = 0.0491), respectively.

The mean DAS28-ESR at each evaluation time point and mean change from baseline to months 6 and 12 are shown in Table 5. The adjusted mean change in DAS28-ESR from baseline to month 6 was − 0.56 (95% CI: − 1.08, − 0.05) in the csDMARDs plus denosumab group and − 0.54 (95% CI: − 1.08, − 0.01) in the csDMARD therapy alone group and that from baseline to month 12 was − 0.79 (95% CI: − 1.32, − 0.26) in the csDMARDs plus denosumab group and − 0.31 (95% CI: − 0.84, 0.23) in the csDMARD therapy alone group.

**Table 5:** Change from baseline in DAS28-ESR results (full analysis set)

	Month	csDMARDs plus denosumab (***N*** = 21)		csDMARD therapy alone (***N*** = 22)		Difference (csDMARDs plus denosumab − csDMARD therapy alone)
		***N***	Adjusted mean [95% CI]	***N***	Adjusted mean [95% CI]	Adjusted mean [95% CI] ***P*** value
**DAS28-ESR**	6	21	− 0.56 [− 1.08, − 0.05]	21	− 0.54 [− 1.08, − 0.01]	− 0.02 [− 0.60, 0.56] *P* = 0.9470
	12	19	− 0.79 [− 1.32, − 0.26]	21	− 0.31 [− 0.84, 0.23]	− 0.48 [− 1.07, 0.11] *P* = 0.1087

Data are shown as adjusted mean [95% CI] unless otherwise indicated

*N* is the number of patients with available data

For adjusted mean, a linear mixed effect model analysis was performed using treatment group, sex, anti-CCP antibody (positive vs negative), baseline DAS28-ESR, measurement time point, and the interaction between the treatment group and measurement time point as fixed effects; patients as random effects; and baseline values as covariates

*CCP* cyclic citrullinated peptide, *CI* confidence interval, *csDMARD* conventional synthetic disease-modifying anti-rheumatic drug, *DAS28-ESR* Disease Activity Score in 28 joints for rheumatoid arthritis with erythrocyte sedimentation rate

Changes in biomarkers are summarized in Additional file 1: Table S4. The between-group difference in the percentage change in procollagen type I N-terminal propeptide levels from baseline to month 12 was − 41.67% (95% CI: − 57.25, − 26.10; *P* < 0.0001); tartrate-resistant acid phosphatase-5b, − 39.50% (95% CI: − 54.36, − 24.64; *P* < 0.0001); and matrix metalloproteinase-3, 3.56% (95% CI: − 112.15, 119.26; *P* = 0.9515).

### Safety

The incidence of AEs and serious AEs were similar between the csDMARDs plus denosumab group and the csDMARD therapy alone group (AEs: 52.2% [12/23] vs 56.5% [13/23], respectively; serious AEs: 4.3% [1/23] vs 8.7% [2/23], respectively) (Table 6). The serious AEs included one event of osteoarthritis in the csDMARDs plus denosumab group and one event each of lung cancer and subdural hematoma in the csDMARD therapy alone group. Six of 23 patients (26.1%) in the csDMARDs plus denosumab group experienced ADRs, the most frequent being hypocalcemia (4/23 patients, 17.4%). Most of the patients recovered without interruption of treatment.

**Table 6:** Safety results (safety analysis set)

	csDMARDs plus denosumab (***N*** = 23)	csDMARD therapy alone (***N*** = 23)
Any AEs	12 (52.2)	13 (56.5)
AEs that occurred in at least two patients in any treatment group		
Herpes zoster	2 (8.7)	0 (0.0)
Nasopharyngitis	1 (4.3)	4 (17.4)
Hypocalcemia	4 (17.4)	1 (4.3)
Hepatic function abnormal	1 (4.3)	2 (8.7)
Liver disorders	0 (0.0)	2 (8.7)
Serious AEs	1 (4.3)^a^	2 (8.7)^b^
Any ADRs	6 (26.1)	0 (0.0)
Hypocalcemia	4 (17.4)	0 (0.0)
Rash erythematous	1 (4.3)	0 (0.0)
Arthralgia	1 (4.3)	0 (0.0)
Muscle spasms	1 (4.3)	0 (0.0)
Pain in extremity	1 (4.3)	0 (0.0)
Malaise	1 (4.3)	0 (0.0)
Platelet count decreased	1 (4.3)	0 (0.0)
Serious ADRs	0 (0.0)	0 (0.0)

According to the Medical Dictionary for Regulatory Activities/Japanese version 23.1

*ADR* adverse drug reaction, *AE* adverse event, *csDMARD* conventional synthetic disease-modifying anti-rheumatic drug

^a^One event of osteoarthritis

^b^One event each of lung cancer and subdural hematoma

## Discussion

This was an exploratory study, and the sample size was set based on the previous randomized controlled trial of denosumab compared with alendronate conducted by Yue et al. [17]. In this open-label, randomized, parallel-group study, 43 patients were included in the FAS (21 and 22 patients in the csDMARDs plus denosumab group and csDMARD therapy alone group, respectively). The results of the primary endpoint showed that the adjusted mean change from baseline in the depth of bone erosion, measured by HR-pQCT, in the 2–3 metacarpal heads at 6 months was − 0.57 mm (95% CI: − 1.52, 0.39 mm) in the csDMARDs plus denosumab group and − 0.22 mm (95% CI: − 0.97, 0.53 mm) in the csDMARD therapy alone group, with a between-group difference of − 0.35 mm (95% CI: − 1.00, 0.31 mm; *P* = 0.2716). Similar results were shown for the adjusted mean between-group difference in the width and volume of bone erosions of the 2–3 metacarpal heads. Significant improvements were observed in bone micro-architecture parameters. The incidences of AEs and serious AEs were similar between the two groups, and no serious AEs were judged as related to denosumab.

The overall background characteristics in this study were generally well-balanced between the treatment groups. However, the proportion of patients who used glucocorticoids was notably higher in the csDMARDs plus denosumab group vs the csDMARD therapy alone group (47.6% vs 18.2%, respectively). As the use of corticosteroids has not been shown to inhibit bone destruction in rheumatoid arthritis, this difference in glucocorticoid use between the groups is considered to have had little effect on the inhibition of bone erosion, although it may have had a small effect on disease activity. Although the csDMARDs plus denosumab group had a higher rate of glucocorticoid use and a lower rate of bisphosphonate use compared with the csDMARD therapy alone group, the predominant improvement in bone micro-architecture in the csDMARDs plus denosumab group may indicate a strong additional concomitant effect of denosumab.

In the present study, a decrease in the depth of bone erosion as measured by HR-pQCT in the 2–3 metacarpal heads from baseline to month 6 was shown in both treatment groups, although there were no statistically significant differences between the groups. The improvement in the depth of bone erosion with denosumab in the present study seemed to be numerically higher than that observed in previous studies: the change from baseline to month 6 in the depth of bone erosion in the denosumab-treated group was − 0.57 mm in the present study and − 0.16 mm at 6 months (*P* <0.01 vs control [alendronate] group) and – 0.06 mm at 24 months (no significance vs placebo) in previous studies by Yue et al. and by So et al. [13, 17]. In the study by Yue et al., not only depth (− 0.16 mm) but also width (− 0.23 mm) and volume (− 0.91 mm^3^) of bone erosion at the second metacarpal head of the nondominant hand was significantly decreased (*P* <0.01) in the denosumab-treated group after 6 months of treatment [17]. Differences in the background characteristics between the study populations, such as race, concomitant DMARD use, or body mass index may have contributed to the difference in the results between the studies. In addition, the study by So et al. included patients with erosions in the 2–4 metacarpal heads, which differs from this study. The lack of a statistically significant difference between the groups in the present study may have been due to the small number of patients with bone erosions in the 2–3 metacarpal heads or the short observation period. Further studies with a larger sample size and longer observation period are needed to confirm whether denosumab treatment added on to conventional treatment is more effective than conventional treatment alone in inhibiting the spread of bone erosion and improving the depth of bone erosion. The lack of bone erosions in the 2–3 metacarpal heads at baseline is also a concern; therefore, future studies are needed to evaluate bone erosions in other locations as well.

In the placebo-controlled study of denosumab by So et al., the authors compared bone erosion in the head of the second to fourth metacarpophalangeal joints by HR-pQCT in RA patients treated with denosumab or placebo for 24 months [13]. The results showed that there was no difference in the changes in bone erosion and joint space parameters at 12 months of treatment. At 24 months of treatment, the percentages of new bone erosions (19% vs 9%, *P* = 0.009) and bone erosion progression (18% vs 8%, *P* = 0.019) were significantly higher in the placebo group vs the denosumab group, while the percentage of bone erosion repair was higher in the denosumab group vs the placebo group (20% vs 6%, *P* = 0.045).

In the present study, the percentage changes from baseline in periarticular vBMD, BV/TV, Tb.Th, and Tb.Sp of the 2–3 metacarpal heads by HR-pQCT were greater in the csDMARDs plus denosumab group compared with the csDMARDs alone group at month 12. These findings suggest that denosumab contributes to the improvement of periarticular bone micro-architecture. We also found that the total bone erosion score of the metacarpal head and wrist joints by MRI was low in the csDMARDs plus denosumab group at months 6 and 12. Additionally, the bone erosion scores at month 12, measured by X-ray and musculoskeletal ultrasound, both tended to decrease from baseline in the csDMARDs plus denosumab group, but tended to increase numerically in the csDMARDs alone group. Furthermore, the study is the first to evaluate in detail the effect of denosumab on the total osteitis score by MRI and PD score by musculoskeletal ultrasound in patients with RA. These inflammation scores increased in both groups from baseline at months 6 and 12, and the between-group difference was not statistically significant in the present study. These results are partially consistent with that of the DESIRABLE study and its follow-up study [9, 18]. In summary, the results suggest that denosumab may be able to contribute to the improvement of bone erosions and bone micro-architecture without affecting inflammation. In Japan, HR-pQCT is not covered by insurance and is not widely used; however, more detailed diagnostic results obtained from HR-pQCT may lead to more appropriate treatment for patients.

Baseline disease activity is a factor associated with the bone erosion score [19, 20], and control of inflammation is important for inhibiting the progression of bone erosion [21]. In this study, we found that denosumab in combination with csDMARDs tended to reduce the progression of bone erosion, although the difference between the groups did not reach statistical significance. These results might support the usefulness of denosumab as an adjunctive therapy as indicated in the 2020 Japan College of Rheumatology clinical practice guideline for RA [22]. Our results also suggest that factors other than intra-articular inflammation (e.g., osteoclast activity) may also contribute to the progression of bone erosion. Our findings are also considered to be consistent with a previous study of Japanese RA patients who had not been previously treated for osteoporosis [23]. In that study, denosumab did not suppress inflammation or RA disease activity but did significantly suppress a marker of bone metabolism. Therefore, denosumab may be beneficial for patients with progressive bone destruction regardless of inflammation status.

The results from our study suggested that periarticular bone micro-architecture was improved by denosumab treatment. The relationship of bone micro-architecture with bone destruction is not fully understood; however, considering that periarticular osteoporosis precedes erosion, and that early periarticular osteoporosis predicts the progression of joint destruction [24–26], it is expected that improving bone micro-architecture may have a subsequent erosion suppression/repair effect.

Although the difference was not statistically significant, the increase in mTSS was smaller in the csDMARDs plus denosumab group compared with the csDMARD therapy alone group at 6 months, while it was larger at 12 months. The joint destruction score of the 2–3 metacarpal heads decreased in the csDMARDs plus denosumab group at both months 6 and 12. In addition, the DAS28-ESR decreased in both groups during the observation period, suggesting that disease activity was well controlled in both groups. Also, only patients with stable disease activity were included in this study. Taken together, these results suggest that the inclusion of patients with well-controlled disease activity may be one reason for the lack of statistically significant improvements in several efficacy endpoints. In this study, an increased joint destruction score of the 2–3 metacarpal heads was observed in the conventional treatment group, even when disease activity was stable. Therefore, it is important to regularly monitor bone erosion regardless of the degree of disease activity.

In the present study, denosumab significantly suppressed bone biomarkers in patients with rheumatoid arthritis, similar to the findings in previous studies [7–9, 23]. This suppression of bone biomarkers may explain the improvement in bone micro-architecture parameters. However, matrix metalloproteinase-3, a cartilage-destroying enzyme, was not suppressed by the addition of denosumab to conventional treatment, suggesting that denosumab may be effective in suppressing bone destruction rather than cartilage destruction.

The safety profile of denosumab was similar to that reported previously [9, 27], and no new safety concerns were raised.

### Limitations

The present study has some limitations, including the relatively short observation period and the heterogeneity of the study population (patients with and without osteoporosis were included). The sample size was calculated based on the number of bone erosions without limiting the site. When planning the study, we assumed that there would be at least one erosion in each of the 2–3 metacarpal heads, but as a result, only eight (38.1%) and 13 (59.1%) patients in the csDMARDs plus denosumab group and csDMARD therapy alone group, respectively, had erosions in the 2–3 metacarpal heads. Based on the original assumption, the number of enrolled patients was sufficient, but the number of patients with erosions in the 2–3 metacarpal heads was smaller than expected, resulting in a smaller sample size. Between-group differences in patient background characteristics may also have influenced the results. For example, regarding the use of csDMARDs, methotrexate was used more frequently in the csDMARDs plus denosumab group than in the csDMARD therapy alone group. Furthermore, smoking has been identified as a factor associated with bone erosion [28]; however, information on smoking history was not collected in this study. In this study, the number of erosive joints available for analysis was small because there were few bone erosions in the joints evaluated. It is possible that if other bone erosions had been evaluated, the differences would have been more apparent. One patient in the csDMARD therapy alone group received denosumab because of erosion progression after registration. Finally, although the results of bone erosion were similar between HR-pQCT and X-ray, the correlation between HR-pQCT and X-ray results was not analyzed using statistical methods.

## Conclusions

In this exploratory study, we investigated the efficacy of denosumab in suppressing the progression of bone erosion in patients with RA being treated with csDMARDs in clinical practice. Although the addition of denosumab to conventional treatment did not lead to a statistically significant improvement in bone erosion, numerical improvements in these parameters suggest that the addition of denosumab to csDMARDs may inhibit the progression of bone erosion. The results also showed improvements in bone micro-architecture parameters, which suggest the benefit of adding denosumab to conventional treatment for improving bone micro-architecture.

## Supplementary Information

**Additional file 1: Table S1.** Measurement values in depth of bone erosion at 6 months by HR-pQCT (full analysis set). **Table S2.** Change from baseline in bone erosion score and JSN score evaluated by X-ray (full analysis set). **Table S3.** Change from baseline in bone micro-architecture parameters evaluated by HR-pQCT, MRI, musculoskeletal ultrasound, and X-ray (full analysis set). **Table S4.** Actual measured values and percent changes from baseline in bone and cartilage biomarkers (full analysis set).

## Data Availability

The datasets supporting the conclusions of this article are available from the corresponding author upon reasonable request.

## Abbreviations

2–3 metacarpal heads: Second and third metacarpal heads
ACR: American College of Rheumatology
ADR: Adverse drug reaction
AE: Adverse event
BMD: Bone mineral density
BV/TV: Trabecular bone volume fraction
CCP: Cyclic citrullinated peptide
CI: Confidence interval
csDMARD: Conventional synthetic disease-modifying anti-rheumatic drug
DAS28-ESR: Disease Activity Score in 28 joints for rheumatoid arthritis with erythrocyte sedimentation rate
EULAR: European Alliance of Associations for Rheumatology
FAS: Full analysis set
HR-pQCT: High-resolution peripheral quantitative computed tomography
JSN: Joint space narrowing
MRI: Magnetic resonance imaging
mTSS: Modified total Sharp score
PD: Power Doppler
RA: Rheumatoid arthritis
RANKL: Receptor activator of nuclear factor-κB ligand
SD: Standard deviation
Tb.Sp: Trabecular separation
Tb.Th: Trabecular thickness
vBMD: Volumetric bone mineral density

## References

[1] Bullock J, Rizvi SAA, Saleh AM, Ahmed SS, Do DP, Ansari RA (2018). Rheumatoid arthritis: a brief overview of the treatment. Med Princ Pract.

[2] Panagopoulos PK, Lambrou GI (2018). Bone erosions in rheumatoid arthritis: recent developments in pathogenesis and therapeutic implications. J Musculoskelet Neuronal Interact.

[3] Tanaka Y (2020). Rheumatoid arthritis. Inflamm Regen.

[4] Kostenuik PJ, Nguyen HQ, McCabe J, Warmington KS, Kurahara C, Sun N (2009). Denosumab, a fully human monoclonal antibody to RANKL, inhibits bone resorption and increases BMD in knock-in mice that express chimeric (murine/human) RANKL. J Bone Miner Res.

[5] Yasuda H, Shima N, Nakagawa N, Yamaguchi K, Kinosaki M, Mochizuki S (1998). Osteoclast differentiation factor is a ligand for osteoprotegerin/osteoclastogenesis-inhibitory factor and is identical to TRANCE/RANKL. Proc Natl Acad Sci U S A.

[6] Zaheer S, LeBoff M, Lewiecki EM (2015). Denosumab for the treatment of osteoporosis. Expert Opin Drug Metab Toxicol.

[7] Cohen SB, Dore RK, Lane NE, Ory PA, Peterfy CG, Sharp JT (2008). Denosumab treatment effects on structural damage, bone mineral density, and bone turnover in rheumatoid arthritis: a twelve-month, multicenter, randomized, double-blind, placebo-controlled, phase II clinical trial. Arthritis Rheum.

[8] Takeuchi T, Tanaka Y, Ishiguro N, Yamanaka H, Yoneda T, Ohira T (2016). Effect of denosumab on Japanese patients with rheumatoid arthritis: a dose-response study of AMG 162 (Denosumab) in patients with RheumatoId arthritis on methotrexate to Validate inhibitory effect on bone Erosion (DRIVE)-a 12-month, multicentre, randomised, double-blind, placebo-controlled, phase II clinical trial. Ann Rheum Dis.

[9] Takeuchi T, Tanaka Y, Soen S, Yamanaka H, Yoneda T, Tanaka S (2019). Effects of the anti-RANKL antibody denosumab on joint structural damage in patients with rheumatoid arthritis treated with conventional synthetic disease-modifying antirheumatic drugs (DESIRABLE study): a randomised, double-blind, placebo-controlled phase 3 trial. Ann Rheum Dis.

[10] Daiichi Sankyo Co., Ltd. Daiichi Sankyo obtains approval for additional indication for PRALIA® subcutaneous injection 60mg syringe. 2017. https://www.daiichisankyo.com/media/press_release/detail/index_3343.html. Accessed 27 April 2022.

[11] Klose-Jensen R, Tse JJ, Keller KK, Barnabe C, Burghardt AJ, Finzel S (2020). High-resolution peripheral quantitative computed tomography for bone evaluation in inflammatory rheumatic disease. Front Med (Lausanne).

[12] Shiraishi K, Chiba K, Watanabe K, Oki N, Iwamoto N, Amano S (2022). Analysis of bone erosions in rheumatoid arthritis using HR-pQCT: development of a measurement algorithm and assessment of longitudinal changes. PLoS One.

[13] So H, Cheng IT, Lau SL, Chow E, Lam T, Hung VW (2021). Effects of RANKL inhibition on promoting healing of bone erosion in rheumatoid arthritis using HR-pQCT: a 2-year, randomised, double-blind, placebo-controlled trial. Ann Rheum Dis.

[14] Iwamoto N, Sato S, Sumiyoshi R, Chiba K, Miyamoto N, Arinaga K (2019). Comparative study of the inhibitory effect on bone erosion progression with denosumab treatment and conventional treatment in rheumatoid arthritis patients: study protocol for an open-label randomized controlled trial by HR-pQCT. Trials..

[15] Aletaha D, Neogi T, Silman AJ, Funovits J, Felson DT, Bingham CO (2010). 2010 rheumatoid arthritis classification criteria: an American College of Rheumatology/European League Against Rheumatism collaborative initiative. Ann Rheum Dis.

[16] Arnett FC, Edworthy SM, Bloch DA, McShane DJ, Fries JF, Cooper NS (1988). The American Rheumatism Association 1987 revised criteria for the classification of rheumatoid arthritis. Arthritis Rheum.

[17] Yue J, Griffith JF, Xiao F, Shi L, Wang D, Shen J (2017). Repair of bone erosion in rheumatoid arthritis by denosumab: a high-resolution peripheral quantitative computed tomography study. Arthritis Care Res.

[18] Tanaka S, Kobayashi M, Saito K, Takita A (2022). Impact of denosumab discontinuation on changes in bone mineral density and bone erosion in rheumatoid arthritis patients. Mod Rheumatol.

[19] Bruno D, Fedele AL, Tolusso B, Barini A, Petricca L, Di Mario C (2021). Systemic bone density at disease onset is associated with joint erosion progression in early naive to treatment rheumatoid arthritis: a prospective 12-month follow-up open-label study. Front Med (Lausanne).

[20] Takeuchi T, Soen S, Ishiguro N, Yamanaka H, Tanaka S, Kobayashi M (2021). Predictors of new bone erosion in rheumatoid arthritis patients receiving conventional synthetic disease-modifying antirheumatic drugs: analysis of data from the DRIVE and DESIRABLE studies. Mod Rheumatol.

[21] Epsley S, Sl T, Farid A, Kargilis D, Mehta S, Rajapakse CS (2021). The effect of inflammation on bone. Front Physiol.

[22] Japan College of Rheumatology (2021). Rheumatoid arthritis clinical practice guidelines 2020.

[23] Kinoshita H, Miyakoshi N, Kashiwagura T, Kasukawa Y, Sugimura Y, Shimada Y (2017). Comparison of the efficacy of denosumab and bisphosphonates for treating secondary osteoporosis in patients with rheumatoid arthritis. Mod Rheumatol.

[24] Hoff M, Haugeberg G, Kvien TK (2007). Hand bone loss as an outcome measure in established rheumatoid arthritis: 2-year observational study comparing cortical and total bone loss. Arthritis Res Ther.

[25] Deodhar AA, Brabyn J, Pande I, Scott DL, Woolf AD (2003). Hand bone densitometry in rheumatoid arthritis, a five year longitudinal study: an outcome measure and a prognostic marker. Ann Rheum Dis.

[26] Güler-Yüksel M, Allaart CF, Goekoop-Ruiterman YP, de Vries-Bouwstra JK, van Groenendael JH, Mallée C (2009). Changes in hand and generalised bone mineral density in patients with recent-onset rheumatoid arthritis. Ann Rheum Dis.

[27] Tanaka Y, Takeuchi T, Soen S, Yamanaka H, Yoneda T, Tanaka S (2021). Effects of denosumab in Japanese patients with rheumatoid arthritis treated with conventional antirheumatic drugs: 36-month extension of a phase III study. J Rheumatol.

[28] Rydell E, Forslind K, Nilsson JÅ, Jacobsson LTH, Turesson C (2018). Smoking, body mass index, disease activity, and the risk of rapid radiographic progression in patients with early rheumatoid arthritis. Arthritis Res Ther.

