# Supplementary material for: Inhibition of bone erosion, determined by high-resolution peripheral quantitative computed tomography (HR-pQCT), in rheumatoid arthritis patients receiving a conventional synthetic disease-modifying anti-rheumatic drug (csDMARD) plus denosumab vs csDMARD therapy alone: an open-label, randomized, parallel-group study
Source: Arthritis Res Ther. 2022 Dec 7;24:264. doi: 10.1186/s13075-022-02957-w (PMC9727996; doi:10.1186/s13075-022-02957-w)
Supplement: Supplementary file 1 — Additional file 1: Table S1. Measurement values in depth of bone erosion at 6 months by HR-pQCT (full analysis set). Table S2. Change from baseline in bone erosion score and JSN score evaluated by X-ray (full analysis set). Table S3. Change from baseline in bone micro-architecture parameters evaluated by HR-pQCT, MRI, musculoskeletal ultrasound, and X-ray (full analysis set). Table S4. Actual measured values and percent changes from baseline in bone and cartilage biomarkers (full analysis set). [file 13075_2022_2957_MOESM1_ESM.docx]

**Additional File**

**Inhibition of bone erosion, determined by high-resolution peripheral quantitative computed tomography (HR-pQCT), in rheumatoid arthritis patients receiving a conventional synthetic disease-modifying anti-rheumatic drug (csDMARD) plus denosumab vs csDMARD therapy alone: An open-label, randomized, parallel-group study**

Naoki Iwamoto^1^*, Ko Chiba^2^, Shuntaro Sato^3^, Kazuteru Shiraishi^2^, Kounosuke Watanabe^2^, Nozomi Oki^4^, Akitomo Okada^5^, Tomohiro Koga^1^, Shin-ya Kawashiri^1, 6^, Mami Tamai^1^, Naoki Hosogaya^3^, Masako Furuyama^7^, Makiko Kobayashi^8^, Kengo Saito^8^, Naoki Okubo^9^, Masataka Uetani^4^, Makoto Osaki^2^, Atsushi Kawakami^1^

**Supplementary Table S1.** Measurement values in depth of bone erosion at 6 months by HR-pQCT (full analysis set)

| **Bone erosion depth of the 2–3 metacarpal heads, mm** | **csDMARDs plus denosumab**  **(N = 21)** | | **csDMARD therapy alone**  **(N = 22)** | |
| --- | --- | --- | --- | --- |
|  | **n** | Mean ± SD | **n** | Mean ± SD |
| Baseline | 18 | 1.57 ± 0.86 | 24 | 1.96 ± 1.21 |
| Month 6 | 17 | 1.70 ± 1.03 | 24 | 2.18 ± 1.37 |
| Change from baseline | 17 | 0.14 ± 0.51 | 24 | 0.22 ± 0.79 |

n is the number of bone erosions.

csDMARD, conventional synthetic disease-modifying anti-rheumatic drug; HR-pQCT, high-resolution peripheral quantitative computed tomography; SD, standard deviation.

**Supplementary Table S2.** Changes from baseline in bone erosion score and JSN score evaluated by X-ray (full analysis set)

|  | **Month** | **csDMARDs plus denosumab**  **(N = 21)** | | **csDMARD therapy alone**  **(N = 22)** | | **Difference (csDMARDs plus denosumab – csDMARD therapy alone)** |
| --- | --- | --- | --- | --- | --- | --- |
|  |  | **N** | **Adjusted mean [95% CI]** | **N** | **Adjusted mean [95% CI]** | **Adjusted mean [95% CI]**  **P value** |
| **Bone erosion score by X-ray, %** | 6 | 15 | −0.50 [−2.79, 1.79] | 19 | −0.45 [−2.54, 1.64] | −0.05 [−2.64, 2.53]  P = 0.9680 |
|  | 12 | 14 | −0.61 [−3.05, 1.84] | 18 | 1.57 [−0.54, 3.68] | −2.18 [−4.85, 0.49]  P = 0.1083 |
| **JSN score by X-ray, %** | 6 | 18 | −1.09 [−10.00, 7.83] | 19 | 4.41 [−4.42, 13.24] | −5.50 [−15.02, 4.02]  P = 0.2505 |
|  | 12 | 17 | −1.02 [−10.16, 8.11] | 18 | 9.19 [0.32,18.06] | −10.21 [−19.88, −0.54]  P = 0.0389 |

Data are shown as adjusted mean [95% CI] unless otherwise indicated.

N is the number of patients with available data.

For adjusted mean, a linear mixed effect model analysis was performed using treatment group, sex, anti-CCP antibody (positive vs negative), baseline DAS28-ESR, measurement time-point, and the interaction between treatment group and measurement time-point as fixed effects; patients as random effects; and baseline values as covariates.

CCP, cyclic citrullinated peptide; CI, confidence interval; csDMARD, conventional synthetic disease-modifying anti-rheumatic drug; DAS28-ESR, Disease Activity Score in 28 joints for rheumatoid arthritis with erythrocyte sedimentation rate; JSN, joint space narrowing.

**Supplementary Table S3.** Changes from baseline in bone micro-architecture parameters evaluated by HR-pQCT, MRI, musculoskeletal ultrasound, and X-ray (full analysis set)

|  | **Month** | **csDMARDs plus denosumab**  **(N = 21)** | | **csDMARD therapy alone**  **(N = 22)** | | **Difference (csDMARDs plus denosumab – csDMARD therapy alone)** |
| --- | --- | --- | --- | --- | --- | --- |
|  |  | n | **Adjusted mean [95% CI]** | n | **Adjusted mean [95% CI]** | **Adjusted mean [95% CI]**  **P value** |
| **vBMD of the 2–3 metacarpal heads by HR-pQCT, mg HA/cm^3^** | 6 | 42 | 6.41 [2.78, 10.03] | 44 | 2.46 [−1.33, 6.24] | 3.95 [−0.05, 7.94]  P = 0.0526 |
|  | 12 | 38 | 9.20 [5.46, 12.95] | 42 | 3.66 [−0.15, 7.46] | 5.55 [1.46, 9.63]  P = 0.0086 |
| **BV/TV of the 2–3 metacarpal heads** **by HR-pQCT, %** | 6 | 42 | 0.93 [0.42, 1.44] | 44 | 0.32 [−0.22, 0.86] | 0.61 [0.04, 1.17]  P = 0.0350 |
|  | 12 | 38 | 1.29 [0.76, 1.82] | 42 | 0.49 [−0.05, 1.03] | 0.80 [0.22, 1.38]  P = 0.0075 |
| **Tb.Th** **of the 2–3 metacarpal heads** **by HR-pQCT, µm** | 6 | 42 | 2.87 [0.08, 5.66] | 44 | 0.97 [−1.90, 3.85] | 1.90 [−1.36, 5.16]  P = 0.2506 |
|  | 12 | 38 | 4.77 [1.80, 7.74] | 42 | 1.93 [−0.97, 4.82] | 2.84 [−0.53, 6.22]  P = 0.0979 |
| **Tb.Sp** **of the 2–3 metacarpal heads** **by HR-pQCT, µm** | 6 | 42 | −12.87 [−33.60, 7.86] | 44 | 6.35 [−15.06, 27.76] | −19.22 [−41.99, 3.55]  P = 0.0963 |
|  | 12 | 38 | −10.39 [−31.84, 11.06] | 42 | 12.44 [−9.08, 33.96] | −22.83 [−46.10, 0.44]  P = 0.0544 |
| **Osteitis score of total metacarpal heads and writs joints by MRI** | 6 | 21^a^ | −0.7 [−3.5, 2.2] | 22^a^ | 0.1 [−2.9, 3.2] | −0.8 [−4.0, 2.4]  P = 0.6125 |
|  | 12 | 20^a^ | 1.4 [−1.6, 4.4] | 21^a^ | −0.5 [−3.6, 2.6] | 1.9 [−1.3, 5.2]  P = 0.2435 |
| **Bone erosion score of total metacarpal heads and wrist joints by MRI** | 6 | 20^a^ | 0.5 [−0.8, 1.8] | 20^a^ | 1.2 [−0.1, 2.6] | −0.7 [−2.1, 0.7]  P = 0.2993 |
|  | 12 | 18^a^ | 1.4 [0.1, 2.8] | 19^a^ | 1.8 [0.5, 3.2] | −0.4 [−1.9, 1.0]  P = 0.5680 |
| **PD score in both hands by musculoskeletal ultrasound** | 6 | 21^a^ | 0.1 [−1.5, 1.6] | 22^a^ | 0.3 [−1.3, 2.0] | −0.3 [−2.1, 1.6]  P = 0.7700 |
|  | 12 | 20^a^ | 0.0 [−1.6, 1.6] | 20^a^ | 0.3 [−1.4, 1.9] | −0.3 [−2.2, 1.6]  P = 0.7853 |
| **Number of bone erosion at both hands by musculoskeletal ultrasound** | 6 | 21^a^ | 0.0 [−0.5, 0.5] | 22^a^ | 0.4 [−0.2, 0.9] | −0.4 [−0.9, 0.2]  P = 0.2030 |
|  | 12 | 20^a^ | 0.0 [−0.5, 0.6] | 21^a^ | 0.6 [0.1, 1.1] | −0.6 [−1.2, 0.0]  P = 0.0491 |
| **Joint destruction score of the 2–3 metacarpal heads by X-ray** | 6 | 42 | −0.03 [−0.11, 0.04] | 44 | 0.02 [−0.05, 0.10] | −0.06 [−0.14, 0.03]  P = 0.1851 |
|  | 12 | 40 | −0.01 [−0.09, 0.07] | 42 | 0.09 [0.01, 0.16] | −0.10 [−0.18, −0.01]  P = 0.0340 |
| **mTSS by X-ray** | 6 | 21^a^ | 0.04 [−0.33, 0.41] | 22^a^ | 0.25 [−0.13, 0.63] | −0.21 [−0.61, 0.19]  P = 0.2962 |
|  | 12 | 20^a^ | 0.12 [−0.26, 0.49] | 21^a^ | 0.49 [0.11, 0.87] | −0.38 [−0.78, 0.03]  P = 0.0659 |
| **Bone erosion score by X-ray** | 6 | 21^a^ | −0.1 [−0.2, 0.0] | 22^a^ | −0.1 [−0.2, 0.0] | 0.0 [−0.1, 0.1]  P = 0.5769 |
|  | 12 | 20^a^ | −0.1 [−0.2, 0.0] | 21^a^ | 0.0 [−0.1, 0.1] | −0.1 [−0.2, 0.0]  P = 0.0972 |
| **JSN score by X-ray** | 6 | 21^a^ | 0.09 [−0.26, 0.45] | 22^a^ | 0.33 [−0.03, 0.70] | −0.24 [−0.62, 0.14]  P = 0.2110 |
|  | 12 | 20^a^ | 0.17 [−0.18, 0.52] | 21^a^ | 0.46 [0.09, 0.82] | −0.28 [−0.67, 0.10]  P = 0.1391 |

Data are shown as adjusted mean [95% CI] unless otherwise indicated.

n is the number of joint evaluated.

^a^N is the number of patients with available data.

For adjusted mean, a linear mixed effect model analysis was performed using treatment group, sex, anti-CCP antibody (positive vs negative), baseline DAS28-ESR, measurement time-point, and the interaction between treatment group and measurement time-point as fixed effects; patients as random effects; and baseline values as covariates.

BV/TV, trabecular bone volume fraction; CCP, cyclic citrullinated peptide; CI, confidence interval; csDMARD, conventional synthetic disease-modifying anti-rheumatic drug; DAS28-ESR, Disease Activity Score in 28 joints for rheumatoid arthritis with erythrocyte sedimentation rate; HR-pQCT, high-resolution peripheral quantitative computed tomography; JSN, joint space narrowing; MRI, magnetic resonance imaging; mTSS, modified total Sharp score; PD, power Doppler; Tb.Sp trabecular separation; Tb.Th, trabecular thickness; vBMD, volumetric bone mineral density.

**Supplementary Table S4.** Actual measured values and percent changes from baseline in bone and cartilage biomarkers (full analysis set)

|  | **Month** | **csDMARDs plus denosumab**  **(N = 21)** | | **csDMARD therapy alone**  **(N = 22)** | | **Difference (csDMARDs plus denosumab − csDMARD therapy alone)** |
| --- | --- | --- | --- | --- | --- | --- |
|  |  | **N** | **Adjusted mean [95% CI]** | **N** | **Adjusted mean [95% CI]** | **Adjusted mean [95% CI]**  **P value** |
| **P1NP, ng/mL** | 6 | 21 | −27.50 [−34.39, −20.62] | 21 | −4.49 [−11.63, 2.65] | −23.01 [−30.74, −15.28]  P <0.0001 |
|  | 12 | 20 | −28.49 [−35.52, −21.46] | 21 | −7.38 [−14.52, −0.24] | −21.11 [−28.88, −13.33]  P <0.0001 |
| **P1NP, %** | 6 | 21 | −54.21 [−68.08, −40.33] | 21 | −7.57 [−21.96, 6.81] | −46.63 [−62.12, −31.15]  P <0.0001 |
|  | 12 | 20 | −54.93 [−69.07, −40.79] | 21 | −13.25 [−27.64, 1.13] | −41.67 [−57.25, −26.10]  P <0.0001 |
| **TRACP-5b, mU/dL** | 6 | 21 | −151.6 [−195.7, −107.5] | 21 | −10.2 [−54.8, 34.4] | −141.4 [−191.7, −91.1]  P <0.0001 |
|  | 12 | 20 | −132.9 [−178.0, −87.8] | 21 | −2.6 [−47.2, 42.0] | −130.3 [−180.9, −79.7]  P <0.0001 |
| **TRACP-5b, %** | 6 | 21 | −41.87 [−54.54, −29.21] | 21 | −0.85 [−13.65, 11.95] | −41.02 [−55.77, −26.28]  P <0.0001 |
|  | 12 | 20 | −37.52 [−50.56, −24.48] | 21 | 1.98 [−10.82, 14.78] | −39.50 [−54.36, −24.64]  P <0.0001 |
| **MMP-3, ng/mL** | 6 | 21 | 8.78 [−41.00, 58.56] | 21 | −7.62 [−59.71, 44.47] | 16.40 [−42.75, 75.55]  P = 0.5836 |
|  | 12 | 20 | −1.38 [−52.91, 50.16] | 21 | −10.16 [−62.24, 41.93] | 8.78 [−51.03, 68.59]  P = 0.7716 |
| **MMP-3, %** | 6 | 21 | 19.31 [−77.18, 115.81] | 21 | −10.45 [−111.45, 90.55] | 29.76 [−84.66, 144.19]  P = 0.6071 |
|  | 12 | 20 | −4.55 [−104.41, 95.32] | 21 | −8.10 [−109.10, 92.90] | 3.56 [−112.15, 119.26]  P = 0.9515 |

Data are shown as adjusted mean [95% CI] unless otherwise indicated.

N is the number of patients with available data.

For adjusted mean, a linear mixed effect model analysis was performed using treatment group, sex, anti-CCP antibody (positive vs negative), baseline DAS28-ESR, measurement time-point, and the interaction between treatment group and measurement time-point as fixed effects; patients as random effects; and baseline values as covariates.

CCP, cyclic citrullinated peptide; CI, confidence interval; csDMARD, conventional synthetic disease-modifying anti-rheumatic drug; DAS28-ESR, Disease Activity Score in 28 joints for rheumatoid arthritis with erythrocyte sedimentation rate; MMP-3, matrix metalloproteinase-3; P1NP, procollagen type I N-terminal propeptide; TRACP-5b, tartrate-resistant acid phosphatase 5b.
